# Validation study of the Chinese Early Development Instrument (CEDI)

**DOI:** 10.1186/1471-2431-13-146

**Published:** 2013-09-23

**Authors:** Patrick Ip, Sophia Ling Li, Nirmala Rao, Sharon Sui Ngan Ng, Winnie Wai Sim Lau, Chun Bong Chow

**Affiliations:** 1 Department of Paediatrics and Adolescent Medicine, Queen Mary Hospital, The University of Hong Kong, Pok Fu Lam, Hong Kong, China; 2 Faculty of Education, The University of Hong Kong, Pok Fu Lam, Hong Kong, China; 3 Department of Early Childhood Education, Hong Kong Institute of Education, Tai Po, Hong Kong, China

**Keywords:** Early Development Instrument, Early child development, Validity, Chinese population, Socioeconomic gradient

## Abstract

**Background:**

The Early Development Instrument (EDI) is a comprehensive instrument used to assess school readiness in preschool children. This study was carried out to evaluate the psychometric properties of the Chinese version of the EDI (CEDI) in Hong Kong.

**Methods:**

One hundred and sixty-seven children were purposefully sampled from kindergartens in two districts with very different socioeconomic statuses. The CEDI was assessed for concurrent validity, internal consistency and test-retest reliability. The developmental vulnerability identified using the CEDI scores was further examined in relation to the socioeconomic status of the district and family.

**Results:**

The CEDI displayed adequate internal consistency, with Cronbach’s alphas ranging from 0.70 to 0.95 on its five domains. Concurrent validity was supported by moderate and significant correlations (0.25 to 0.49) on the relevant domains between the CEDI and a comparable measure. The level of test-retest reliability was good, with a kappa statistic of 0.89. In general, girls outperformed boys, particularly in the social, emotional and communication/general knowledge domains. After controlling for the uneven distribution of sex, children from socioeconomically disadvantaged districts and families were found to be at greater risk of developmental vulnerability than their more advantaged counterparts.

**Conclusion:**

The evidence gathered in this study supports the CEDI’s use as a valid and reliable instrument in assessing school readiness and identifying developmentally vulnerable children in Chinese populations. Its preliminary findings on the socioeconomic gradients of child development suggest that the CEDI is a promising tool for leveraging evidence-based, context-sensitive policies and practices to foster the development of all children.

## Background

Early childhood development is the foundation of human and community development [1]. The early years of life are a critical developmental period for both resilience and vulnerability [2]. School readiness among preschool children has become an important concern for educators, academics and policy-makers [3]. Rather than focus on standard test scores and cognitive capabilities, the Early Development Instrument (EDI), which was developed in Canada by Janus and Offord in 2007 [4], is a comprehensive teacher-completed instrument that assesses school readiness. It covers five major developmental domains, including physical health and well-being, social competence, emotional maturity, language and cognitive development, and communication skills and general knowledge.

Research shows the EDI to be a valid, reliable and stable measure [5-7], and to have small to moderate levels of association with other teacher-reported measures. Studies show its internal consistency to be high, ranging from 0.84 to 0.96, and inter-rater reliability to be satisfactory, ranging from 0.53 to 0.80. Janus et al. (2007) reported the test-retest correlation of the EDI administered twice to the same group of children within a reasonable period of time to be high (0.82 to 0.94) [8], and there is also evidence of its predictive validity for primary school performance when administered during kindergarten [9].

Although the EDI is reliable at the individual level, one of its strengths is to allow the aggregation of individual data to the group or community level, thus permitting examination of the role of socioeconomic inequalities in child development from multiple perspectives [10-13]. Mapping the socioeconomic inequality patterns in early child development can aid communities and countries in forming universal and targeted policies to improve outcomes for all children [14,15].

The aim of this study was to examine the internal consistency, concurrent validity and test-retest reliability of the Chinese Early Development Instrument (CEDI). The CEDI data were also analyzed in relation to socioeconomic indicators to explore the potential existence of socioeconomic disparities in child development among preschoolers in a Chinese community.

## Methods

### Participants and procedures

In 2011, four Chinese-speaking kindergartens were randomly selected from Hong Kong Island (HKI) and Yuen Long District (YL), two major administrative districts in Hong Kong with dramatically different economic levels. HKI is a wealthy district with median monthly family income of around US$4240, which is 33.2% higher than the population average, whereas the corresponding figure for YL is around US$2680, 15.7% below the population average [16]. Ethical approval for this study was granted by the ethics committee of the University of Hong Kong.

All four kindergartens contacted agreed to join the study. With the approval of their principals, all third-year kindergarten (K3) children and their teachers and parents were invited to participate. In total, 175 children were contacted, and 167 K3 children were assessed with both the Chinese Early Development Instrument (CEDI) and the Hong Kong Early Child Development Scale (HKECDS). Informed written consent was obtained from the parents of all participating children. Of these children, 15 from each district were then re-assessed with the CEDI by the same teacher four weeks later to evaluate the instrument’s test-retest reliability. The teacher who was most familiar with each child was invited to rate him or her with the CEDI. To minimize measurement errors introduced by different raters, all of the teachers were trained beforehand in the instrument’s use. This training took the form of two three-hour workshops with home exercises assigned in between. The teachers were given a Chinese version of the CEDI teacher’s guide, which is a comprehensive and user-friendly reference book on the instrument’s use, coding and interpretations of items in the local context. The HKECDS results were assessed by a separate team of trained research assistants with no knowledge of the CEDI results, and the children’s parents were asked to complete a family questionnaire (FQ). The completed CEDI, HKECDS and FQ were collected by the research team.

### Measures and variables

#### Chinese early development instrument (CEDI)

The CEDI was translated from English into Traditional Chinese with the permission of the EDI authors [4] using the back-translation method to translate and adapt the assessment instrument in a trans-cultural context [17]. A bilingual local university faculty member specializing in early childhood education translated the original English-language version into traditional Chinese. Another bilingual faculty member from the same department then translated it back into English separately. Local experts in child development, including university faculty, pediatricians, kindergarten teachers and education experts, were consulted on the local relevance of the instrument’s items, as well as the appropriateness and accuracy of their wording. Three items referring to English letters within the language and cognitive development domain required further modification to fit the context of the learning and use of Chinese characters. The finalized CEDI was then sent to the EDI authors at the Offord Centre for Child Studies (in Hamilton, ON, Canada) for review, and their approval was subsequently obtained.

Consistent with the EDI, the CEDI is also made up of 103 items assessing five developmental domains: a) physical health and wellbeing, b) social competence, c) emotional maturity, d) language and cognitive development, and e) communication skills and general knowledge. The domain scores range from 0 to 10, and the items reflect children’s developmental milestones rather than specific curriculum goals. Children who score in the bottom 10th percentile in at least one of the five domains are deemed *vulnerable* in terms of school readiness, indicating that the problems they have within a given developmental area are likely to interfere with their success in school. The most recent evidence from the longitudinal study in Australia suggested that the *vulnerability* at school entry predicts the literacy and numeracy outcomes throughout primary school years [18].

#### Hong Kong early child development scale (HKECDS)

The HKECDS is a direct assessment of child development (at 3–6 years) that was developed in Hong Kong and shown to display satisfactory psychometric qualities and excellent cultural and contextual appropriateness [19]. The scale contains 95 items in eight domains: a) personal, social and self-care; b) language development; c) pre-academic learning; d) cognitive development; e) gross motor; f) fine motor; g) physical fitness, health and safety (knowledge about); and h) self and society. Compared to the CEDI domain structure, the HKECDS places greater emphasis on knowledge and learning and less on social and emotional assessment. Therefore, we expected the conceptually comparable domains between the two measures (CEDI *with* HKECDS) to be: a) the physical and well-being domain *with* gross and fine motor ability; b) language and cognitive development *with* language, pre-academic learning, and cognitive development; and c) communication skills and general knowledge *with* language and cognitive development and self and society. However, none of the HKEDCS domains specifically matches the social and emotional domain of the CEDI. The concurrent validity of the CEDI was assessed by its correlations with the HKECDS.

#### Family questionnaire (FQ)

Information on the socioeconomic background of the participating children was obtained from their parents using the FQ, a self-developed, pre-tested questionnaire. Maternal education was measured with a single item on a scale ranging from 1 to 7, with higher scores representing higher education levels. In analysis of this study, maternal education was divided into three categories: junior secondary education and below was defined as “low”, senior secondary education to an associate degree as “medium” and a Bachelor’s degree and above as “high”. Family income was measured with one item soliciting total monthly family income on a scale ranging from 1 to 10 (from < HK$4000 to > HK$80,000) (US$1 ≈ HK$7.8). With reference to Hong Kong’s family income distribution in 2011 [16], family income was further categorized into four context-meaningful groups: < $8000 was deemed the lowest 10th percentile of family income distribution; $8000 ~ < $20,000 was below the population median ($22,000); 20,000 ~ < 80,000 covered the population median and the majority of the top half; and >= $80,000 was the highest 10th percentile.

### Data analysis

Because of the uneven sex distribution between the sampled districts, with many more girls in the HKI sample than the YL sample, statistical adjustment was adopted in the following analyses wherever appropriate. Concurrent validity was assessed using the partial correlations between the CEDI and HKECDS domain scores, with sex controlled. Because the two instruments differed in their conceptual structure of child development measurement, the two best correlation coefficients were highlighted in the correlation matrix. Internal consistency was calculated using Cronbach’s α for each of the five CEDI domains. The test-retest reliability of the two scales was determined using the kappa statistic (k). The relationship between child development vulnerabilities and socioeconomic indicators (district, family income and maternal education) was measured by the adjusted odds ratios from logistic regressions after controlling for sex. Statistical analysis was performed using SPSS (version 17), and *p*<0.05 was considered statistically significant.

## Results

### Characteristics of subjects

Of the 167 children who participated in the study, seven were excluded from analysis, four of them because of a special needs designation and three because of missing data on one or more domains. In view of the wide age range of the remaining 160 children (5.43 to 7.31 years), we further restrained our analysis to children born in 2005, which resulted in 151 children in the same age cohort. Table 1 summarizes the subjects’ characteristics. Sixty-six (43.7%) children were from HKI (the wealthy district) and 85 (56.3%) from YL (the poor district). Because the children in the HKI kindergartens were predominately female, the sex distribution of our sample is severely imbalanced, with two-thirds of the subjects girls. The samples from the two districts also differed significantly in terms of the socioeconomic indicators of maternal education and family income.

**Table 1:** Major socioeconomic characteristics of subjects

	**N (%)**	**HKI (%)**	**YL (%)**	***p*-value**
Male	52 (34.4)	10 (15.2)	42 (49.4)	
Females	99 (65.6)	56 (84.8)	43 (50.6)	< 0.001
Maternal Education^^^				
Low	13 (8.8)	0	13 (15.9)	< 0.001
Medium	95 (64.2)	29 (43.9)	66 (80.5)	
High	40 (27.0)	37 (56.1)	3 (3.7)	
Family Income (HK$)				< 0.001
< 8000	18 (12.2)	0	18 (22.2)	
8000 ~ < 20,000	49 (33.3)	2 (3.0)	47 (58.0)	
20,000 ~< 80,000	60 (40.8)	45 (68.2)	15 (18.5)	
>= 80,000 > 80,000	20 (13.6)	19 (28.8)	1 (1.2)	

Note: ^ Maternal education was categorized into three levels: low = junior secondary education and below; medium = senior secondary education to associate degree; and high = Bachelor’s degree and above. The unequal sample size is due to missing data.

### Internal consistency

Cronbach’s α, a measure of internal consistency, ranges from 0.70 to 0.95 for the five CEDI domains (Table 2).

**Table 2:** Summary of domain scores and internal consistency with Cronbach’s α

**Domain**	**Items**	**Mean**	**sd**	**Min**	**Max**	**Cronbach’s α**
1. Physical health and well-being	13	8.77	1.19	3.46	10.00	0.70
2. Social competence	26	8.04	1.71	2.69	10.00	0.95
3. Emotional maturity	30	7.91	1.33	3.67	10.00	0.91
4. Language and cognitive development	26	8.97	1.52	3.20	10.00	0.90
5. Communication skills and general knowledge	8	8.07	2.07	1.88	10.00	0.91

### Concurrent validity

Based on the partial correlations with sex controlled, Table 3 highlights the two strongest correlations with the HKECDS for each CEDI domain. As expected, the physical health and well-being domain correlates best with gross and fine motor, language and cognitive development with pre-academic learning and language development, and communication and general knowledge with language and cognitive development. Because no HKECDS domain specifically measures social and emotional development, the social competence and emotional maturity domains of the CEDI were found to correlate best with gross motor and language development.

**Table 3:** Partial correlations between CEDI and HKECDS domain scores, with sex as the control variable

**CEDI**	**P**	**S**	**E**	**L/C**	**C/G**
**HKECDS**					
Gross Motor	.30**	.41***	.31***	.13	.26*
Fine Motor	.25**	.32***	.11	.25**	.23*
Language Development	.16	.35***	.32***	.47***	.37***
Pre-academic Learning	.22*	.22*	.30***	.49***	.19*
Cognitive Development	.17	.27**	.27**	.39***	.28**
Personal, Social, Self-care Environment	.19*	.19*	.20*	.32***	.21*
Self and Society	.21*	.32***	.26**	.43***	.27**
Physical Fitness, Health and Safety (*Knowledge about…*)	.09	.20*	.24**	.41***	.23**

Note: For the CEDI domains: P = Physical Health and Well-being; S = Social Competence; E = Emotional Maturation; L/C = Language and Cognitive Development; and C/G = Communication and General Knowledge; ***p < .001, **p < .01 and *p < .05.

### Reliability

The test-retest reliability of the CEDI after a four-week interval was analyzed in 30 participants using the kappa statistic (k). The kappa coefficient was 0.89, thus demonstrating the instrument’s stability over time.

### Vulnerability

The cut-offs for vulnerability derived from our sample in Hong Kong are largely comparable with the Canadian normative references in the physical, social and emotional domains, but higher in the language/cognitive and communication/general knowledge domains [8]. As shown in Table 4, 28.5% of the children in our study were found to be developmentally vulnerable in at least one CEDI domain, and 13.9% in at least two. Further, significantly more boys than girls (46.2% boys versus 19.2% girls) were identified as vulnerable (*p* < 0.05) in at least one developmental domain.

**Table 4:** Cut-offs for vulnerability and distribution by sex

		**CEDI**				
	**P**	**S**	**E**	**L/C**	**C/G**	**Total**
Cut-offs						
HK	6.9567	5.3846	6.0000	6.4400	5.0000	
Canada	7.0833	5.5769	6.0000	5.7692	4.3750	
Vulnerability N (%)						
	16 (10.6)	15 (9.9)	16 (10.6)	16 (10.6)	23 (15.2)	43 (28.5)
Male	9 (17.3)	12 (23.1)	10 (19.2)	8 (15.4)	14 (26.9)	24 (46.2)
Female	7 (7.1)	3 (3.0)^***^	6 (6.1)^*^	8 (8.1)	9 (9.1)^***^	19 (19.2)^***^

Note: For the CEDI domains: P = Physical Health and Well-being; S = Social Competence; E = Emotional Maturation; L/C = Language and Cognitive Development; and C/G = Communication and General Knowledge; ***p < .001, **p < .01 and *p < .05.

### Relationship with socioeconomic status of district and family

#### District

Comparison of the socioeconomic status of the two communities in which the participating kindergartens were located showed a significantly higher proportion of children from the socioeconomically disadvantaged district, YL (42.4%), to display developmental vulnerability in at least one of the CEDI domains relative to their HKI counterparts (16.3%). After adjusting for the uneven distribution of sex in our sample, the excessive risk of vulnerability for the YL children still remained significant (aOR = 4.46, 95% CI: 1.74-11.41; *p* < 0.005).

#### Family income

**Figure 1:**
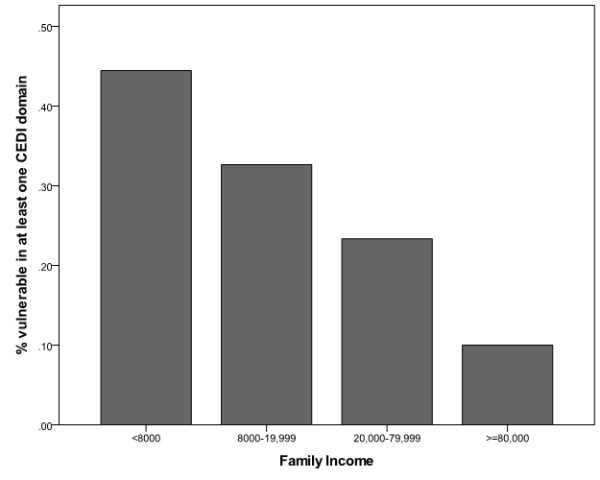
Developmental vulnerability versus family income.

Investigation of the relationship between a vulnerable classification in one or more developmental domains and family income revealed a decreasing gradient (see Table 5 and Figure 1), indicating that children from poorer families are at greater risk of developmental vulnerability than those from relatively wealthy families. After taking the uneven sex distribution into account, the gradient trend between vulnerability and family income remained, as shown in the decreasing adjusted odds ratio with increasing family income in Table 5, although the relationship was no longer statistically significant because of the reduced sample size.

**Table 5:** Descriptive statistics for the CEDI domain scores and vulnerability by family income level

**Family income**	**CEDI domain scores – mean (sd)**					**Vulnerability**	**aOR**^**#**^
	**P**	**S**	**E**	**L/C**	**C/G**	**N (%)**	**[95% CI]**
< 8000	8.57	7.74	7.61	8.27	7.43	8	4.88
	(1.21)	(1.73)	(1.30)	(1.88)	(2.77)	(44.4)	[.82, 28.89]
8000 ~ < 20,000	8.36	7.80	7.68	8.75	8.10	16	2.36
	(1.41)	(1.72)	(1.10)	(1.42)	(2.21)	(32.6)	[.45, 12.35]
20,000 ~ < 80,000	9.09	8.17	7.95	9.18	8.46	14	1.98
	(0.97)	(1.72)	(1.52)	(1.43)	(1.81)	(23.3)	[.39, 9.34]
>= 80,000	8.73	8.56	8.41	9.62	7.47	2	1
	(1.11)	(1.54)	(1.14)	(0.85)	(1.62)	(10.0)	

Note: For the CEDI domains: P = Physical Health and Well-being; S = Social Competence; E = Emotional Maturation; L/C = Language and Cognitive Development; and C/G=Communication and General Knowledge; #odds ratio estimated in the logistic regression with sex adjusted.

#### Maternal education

**Figure 2:**
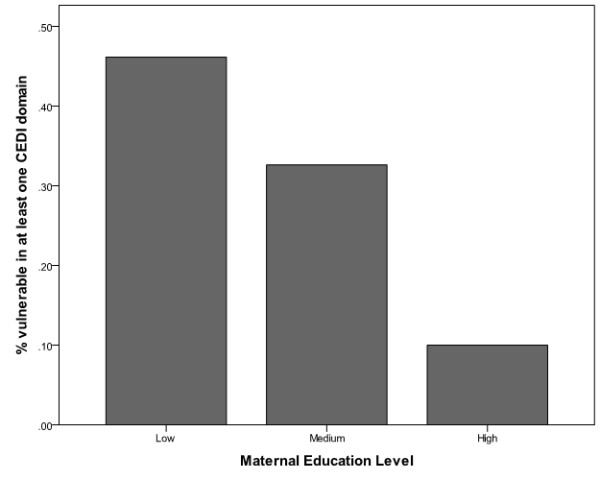
Developmental vulnerability versus maternal education level.

Across all of the CEDI domains, a decreasing gradient can be seen in the mean of the domain scores with maternal education level (see Table 6). The lowest mean scores were found in the group of children whose mothers had a junior secondary level of education or less, whereas the highest scores were found in the group whose mothers held a Bachelor’s degree or higher academic qualification. A similar decreasing gradient with maternal education level was also found in the proportion of children identified as vulnerable in one or more developmental domains (see Table 6 and Figure 2). After controlling for the effect of sex, the gradient trend between vulnerability and maternal education remained significant (*p*<0.05), as illustrated in the decreasing adjusted odds ratios with higher maternal education shown in Table 6.

**Table 6:** Descriptive statistics for CEDI domain scores and vulnerability by maternal education level

**Maternal education**	**CEDI domain score – mean (sd)**					**Vulnerability**	**aOR**^**#**^
	**P**	**S**	**E**	**L/C**	**C/G**	**N (%)**	**[95% CI]**
Low	8.38 (1.10)	7.88	7.65	8.35	7.88	18	5.39*
		(1.72)	(1.19)	(1.98)	(2.46)	(37.5)	[1.14, 25.46]
Medium	8.85	8.10	7.91	9.18	8.11	23	3.41*
	(1.26)	(1.75)	(1.40)	(1.22)	(1.91)	(25.8)	[1.09, 10.73]
High	9.29	8.18	8.66	9.58	8.58	0	1
	(0.89)	(1.47)	(0.80)	(0.73)	(1.48)	(0)	

Note: Low = a maternal education level of junior secondary and below; medium = senior secondary education or a higher certificate or diploma; and high = a Bachelor’s degree and above. For the CEDI domains: P = Physical Health and Well-being; S = Social Competence; E = Emotional Maturation; L/C = Language and Cognitive Development; and C/G = Communication and General Knowledge. #Odds ratio estimated in the logistic regression with sex included; *p < .05, **p < .01 and ***p < .005.

## Discussion

This study examined the internal consistency, concurrent validity and reliability of the Chinese Early Development Instrument (CEDI), which was adapted from the EDI [4]. CEDI is a population tool to assess children’s development at aggregate level and it is not mean to assess children’s school readiness at the individual level. The preliminary evidence obtained therein supports the CEDI’s use as a valid and reliable measure of early child development and school readiness in Chinese populations.

The internal consistency of the five CEDI domains ranged from 0.70 to 0.95, which is comparable with that of the EDI domains [4]. As a Cronbach’s α ranging between 0.70 to 0.90 is generally considered good [20], we can conclude that the CEDI domains demonstrate an adequate level of internal consistency. The test-retest reliability of the CEDI was also found to be good (0.89). These two psychometrical properties of the CEDI are largely comparable with those of the EDI when used in English-speaking countries [7,8].

The CEDI’s concurrent validity was established through comparison with the Hong Kong Early Child Development Scale (HKECDS) [19], a direct assessment of early childhood development developed locally in Hong Kong. After controlling for sex, strong and significant correlations remained between the CEDI and HKECDS in the expected domains. The correlation coefficients (0.25 to 0.49) were comparable to those reported between the EDI and direct child-based assessment, which ranged from 0.34 to 0.49 [21]. The moderate correlations in the current study were expected because the comparison was between a teacher evaluation (CEDI) and direct child-based assessment (HKECDS) across a wide range of differently categorized domains. Stronger correlations have been reported in studies comparing the EDI with other teacher-rated measures [21], although such comparisons are often subject to the problem of shared method variance [22].

In addition, although lacking pre-specified correlates, the social and emotional domains of the CEDI were found to correlate best with the gross motor and language domains of the HKECDS. Children with advanced motor development may display more constructive engagement in early activities, and thus have a better chance of acquiring key social and emotional abilities, and vice versa [23,24]. Similarly, children experiencing delayed language development are likely to find it more difficult to acquire appropriate social and emotional skills [25,26].

In this study, vulnerability was defined according to our Hong Kong sample rather than using Canadian normative data. Although doing so undoubtedly introduced bias, given the small sample size and non-representative sampling structure, the value of using cut-offs from a local sample is that there are recognized differences between the Canadian and Hong Kong Chinese populations with regard to the cultural and developmental context of preschool children, including societal expectations, parenting and the kindergarten environment. Further examination of the relationship between socioeconomic disadvantage and developmental vulnerability revealed the children from an underprivileged district (Yuen Long [YL]) and family (as measured by family income, and maternal education) to be at greater risk of vulnerability in one or more developmental domains. Observations with the CEDI in the Hong Kong Chinese population are consistent with EDI observations in Western societies [11,13].

### Limitations

This study suffered several limitations. First, its main limitation lies in recruitment of the sample. Though kindergartens were randomly selected from Hong Kong Island (HKI) and Yuen Long (YL), the HKI sample included significantly more girls than boys, and the reverse was true for the YL sample, because of the natural sex composition of the kindergartens recruited. Although this severe sex imbalance may not have posed serious harm in testing the psychometric properties of the CEDI, as the EDI factor structure has been reported stable between boys and girls [4], quantitative interpretations of the coefficients should be made with caution. To account for the compound effect between the imbalanced sex distribution and the difference in the socioeconomic status of the two districts, analyses between the CEDI and other factors were statistically adjusted for sex. Second, the EDI is intended for use and interpretation at the group level, whereas the current validation of the CEDI was conducted at the individual level. Third, because of the relatively small sample size, confirmatory factor analysis was not conducted in this study. Fourth, the CEDI questionnaire was completed by the kindergarten teacher who was most familiar with each tested child, and we did not repeat the test with a different teacher; therefore, inter-rater reliability was not assessed.

## Conclusion

In conclusion, the evidence presented herein shows the CEDI to be a psychometrically sound measurement tool for early child development and the assessment of school readiness in Chinese populations. As the EDI has gained significant international popularity in the past decade, with successful adaptation and application in 23 countries, this validation study opens up the exciting possibility of placing Chinese children’s development on an international scale for comparison.

## Competing interests

The authors declare that they have no competing interests.

## Authors’ contributions

PI designed the study, interpreted the data and wrote the manuscript. SLL analyzed the data and drafted the manuscript. NR participated in preparation of assessment tools and interpretation of data. SSNN participated in training of teachers and preparation of assessment tools. WWSL participated in training of teachers and data collection. CBC participated in research design and data interpretation. All authors read and approved the final manuscript.

## Pre-publication history

The pre-publication history for this paper can be accessed here:

http://www.biomedcentral.com/1471-2431/13/146/prepub
